# Short Report: Asymptomatic Zika virus infections with low viral loads not likely to establish transmission in New Orleans *Aedes* populations

**DOI:** 10.1371/journal.pone.0233309

**Published:** 2020-05-29

**Authors:** Matthew J. Ward, Brendan H. Carter, Christine E. S. Walsh, Joshua O. Yukich, Dawn M. Wesson, Rebecca C. Christofferson

**Affiliations:** 1 Department of Tropical Medicine, Tulane University School of Public Health and Tropical Medicine, New Orleans, Louisiana, United States of America; 2 Institute for Global Health and Infectious Disease, University of North Carolina at Chapel Hill, Chapel Hill, North Carolina, United States of America; 3 Department of Pathobiological Sciences, School of Veterinary Medicine, Louisiana State University, Baton Rouge, Louisiana, United States of America; 4 Center for Applied Malaria Research and Evaluation, Tulane University School of Public Health and Tropical Medicine, New Orleans, Louisiana, United States of America; 5 Center for Computation and Technology, Louisiana State University, Baton Rouge, Louisiana, United States of America; Faculty of Science, Ain Shams University (ASU), EGYPT

## Abstract

*Aedes aegypti* and *Aedes albopictus* are both vectors of Zika virus and both are endemic to the New Orleans Metropolitan area. Fortunately, to date there has been no known autochthonous transmission of Zika virus in New Orleans. No studies of the vector competence of local populations of *Ae*. *aegypti* and *Ae*. *albopictus* for Zika virus transmission have been conducted. To determine if New Orleans *Ae*. *aegypti* and *Ae*. *albopictus* mosquitoes are competent for Zika virus, mosquitoes were reared to generation F3 from eggs collected in New Orleans during the 2018 mosquito season. Adults were fed an infectious blood meal and kept for 15 days in an environmental chamber. Transmission assays were conducted at 4, 10, and 15 days post exposure and RT-PCR was run on bodies and saliva to detect the presence of Zika virus RNA. We observed remarkably low susceptibility of both *Ae*. *aegypti* and *Ae*. *albopictus* from New Orleans to a Zika strain from Panama after oral challenge. These results suggest a limited risk of Zika virus transmission should it be introduced to the New Orleans area, and may partially explain why no transmission was detected in Louisiana during the 2016 epidemic in the Americas, despite multiple known travel associated introductions to New Orleans. Despite these results these mosquito populations are known to be competent vectors for some other mosquito-borne viruses and control measures should not be relaxed.

## Introduction

Arboviruses are an ongoing concern for regions where their vectors are endemic, namely most tropical and subtropical areas. Factors such as global warming and globalization have enabled the vectors of these viruses to spread into new geographical regions and support autochthonous transmission of a number of human disease causing arboviruses [1]. Similarly, easy global travel has allowed people and potential reservoirs of these diseases to quickly crisscross the globe, connecting virus to vector [1]. The effects of these and other factors have been made particularly obvious with the recent emergence and subsequent epidemic of Zika virus in the Americas and Caribbean during 2015 [2]. The factors that enable these emergences are not likely to disappear in the near future, and it is therefore reasonable to conclude that future emerging arboviruses and epidemics similar to 2015 are unavoidable.

Zika virus is a flavivirus first detected in the Americas in March 2015 when it was diagnosed in Bahia, Brazil, although it is thought to have been in circulating since at least February of that year [2–5]. Until its emergence in Brazil, Zika virus outbreaks had been limited to small populations resulting in proportional outbreaks. However, when Zika virus emerged in the Western Hemisphere, the presence of a competent vector and a largely immunologically naïve population resulted in a large-scale epidemic that affected most of the western tropics. Its association with neuropathology had also not been recorded [6–9]. As demonstrated by the confusion in diagnosis, Zika’s typical pathology is that of a non-descript febrile illness that was not easily differentiable from that of mild dengue or most other febrile illnesses. By early 2016, an association between clusters of cases of micro-cephalic neonates and nearly 7,000 reported cases of Zika virus was made. On February 1, 2016 the World Health Organization declared that Zika constituted a Public Health Emergency of International Concern [9]. Despite this, Zika virus and microcephaly continued to spread to Brazil’s neighbors and Northward to the Caribbean and Central America culminating with its most northerly autochthonous transmission in Miami, Florida [9].

Viremia levels of Zika patients vary according to several factors. For instance, one study found that hospitalized patients in Nicaragua had higher viremia compared to non-hospitalized patients [10]. In this study, the non-hospitalized patients got up to 4.1 log virus/mL, and it could be presumed that non-hospitalized patients have more contact with mosquitoes compared to those in a hospital where mosquito avoidance may be employed. Other studies have also showed low-level viremia in the range of 4 logs/ml in asymptomatic or subclinical patients [11, 12]. Thus, these low-viremia patients represent an opportunity for transmission if they come into contact with competent vectors.

*Aedes aegypti* and *Ae*. *albopictus* are both competent vectors for Zika virus and both are endemic to the New Orleans Metropolitan area [13, 14]. To date, there has been no local transmission of Zika virus in New Orleans, despite opportunities for introduction via returning travelers with presumed transmissible levels of viremia [15]. We determined whether New Orleans mosquitoes were competent for potential transmission of Zika virus following the hypothetical introduction of asymptomatic or subclinical cases.

## Methods

### Mosquito rearing

Mosquito eggs were collected on seed germination paper (Anchor Paper, St. Paul, MN) lining the inside of oviposition cups placed at sites throughout the New Orleans area during the 2018 season. Collections were on both private and public lands with consent of homeowners, and consent was not needed for public land. No endangered or protected species were involved in this study. Eggs were hatched, reared to adults, and the adults were identified to species [15, 16]. *Aedes aegypti* and *Ae*. *albopictus* colonies were reared to F3. Larvae were reared in square 4 L 9 in. x 7 in. Nalgene pans at a density of 200 larvae per pan in 2.5 L of autoclaved deionized water and fed a 2:3 mixture of brewer’s yeast and liver powder. Adults were housed in 14 in. x 14 in. white PVC cages (BioQuip, Rancho Dominguez, CA) and maintained on 10% sucrose solution and human donor blood (The Blood Center, New Orleans, LA) in an insectary at 27 °C and 80% relative humidity (RH) with a 10 h D:14 h L photoperiod.

### Power calculation and sample size determination

A Monte Carlo simulation was used to produce a power curve, by generating ten-thousand simulated data sets with expected proportions of infection for sampling days 5, 7, 9, and 14. Each simulated data set was analyzed using a generalized linear model with a logistic link function and binomial likelihood. The following expected proportions of infection at each sampling point for each species were as follows for days 5, 7, 9 and 14 respectively: *Ae*. *aegypti–* 0%, 30%, 40%, 60% and *Ae*. *albopictus–* 0%, 3%, 13%, 33%. *Aedes aegypti* proportions were derived from work previously published by Christofferson and Mores (2011) on *Ae*. *aegypti* competence for dengue virus and the *Ae*. *albopictus* proportions were based on unpublished preliminary data using Zika-MEX [17]. If the glm applied to each simulated dataset generated a *p*-values < = 0.05 were the simulated experiment was considered to have detected the true difference between species and was therefore considered as having sufficient power, the power of detecting a significant difference between species at each time point was calculated by dividing the number of these results by the total number of simulations for each simulated sample size and sampling point to provide an estimate of power given alpha of 0.05.

Sample sizes of 10, 12, 14, 16, 18 and 20 females were tested using the simulation and yielded powers of 67%, 76%, 82%, 87%, 91%, and 93%. The simulations and power calculation were carried out using RStudio version 1.1.463 and the ggplot2, scales, knitr, rmarkdown and pwr packages [18–23].

### Exposure

Oral challenge was carried out for both *Ae*. *aegypti* and *Ae*. *albopictus* at a dose of 4 logs PFU/mL. Females between 2 and 4 days post-eclosion were sorted by aspiration into groups with 10 males each and placed in an environmental chamber at 27 °C and 80% relative humidity on a 15-step (14 hr L: 10 hr D) photoperiod 24 hours prior to oral challenge. Sucrose was withheld for 12 hours prior to feeding. A Hemotek membrane feeding system (Hemotek Ltd, Great Harwood, UK) set to 37°C covered with Hemotek’s collagen membrane was used. Infectious blood-meals were prepared using a mixture of washed, deactivated and defibrinated bovine blood (Lampire Biological Laboratories Inc, Pipersville, PA) and centrifuged infectious cell media at a 1:1 ratio. Additionally, ATP was added as a feeding stimulant to a final concentration of 5 μM in the 12 mL prepared meal [24]. Infectious media was taken from fresh Vero cell culture of Zika-Panama (PA259249) (GenBank accession # KX156775) obtained from the University of Texas Medical Branch at culture days 3 and 4 and Vero passage 6. A corresponding plaque assay was run on the infectious media to determine the viral titer of each infectious blood-meal (S1 Table). Mosquitoes were allowed to feed for approximately 1 hour. Post-feeding mosquitoes were cold anesthetized and sorted and engorged females of each species were divided into four groups and replaced in environmental chambers. Mosquitoes were provided 10% sucrose solution on saturated cotton balls for sustenance.

### Sample collection

Prior to the salivation assay, mosquitoes were starved off sucrose for 12 hours. For the low dose experiment, mosquitoes were force salivated at 4, 10, and 15 DPI. Briefly, females were anesthetized with triethylamine in a sealed container (Thermo Fisher Scientific, Waltham, MA) [25]. Legs and wings were removed and placed in 2 mL round-bottom tubes and frozen at -80 °C with a 4 mm stainless steel ball bearing. Mosquitoes were placed on inverted autoclave tape on a salivation plate with proboscises inserted into a 20 μL micro-pipette tip containing 10 μL of salivation solution: 10% sucrose/10% FBS/5 μM ATP [24]. Mosquitoes were allowed to salivate for at least one hour [26, 27]. The contents of each micro-pipette tip were ejected into 2 mL round bottom tubes with 90 uL BA-1. Heads and bodies were placed in 2 mL round bottom tubes with a 4 mm stainless steel ball bearing. Samples were immediately frozen at -80 °C until further processing.

### Zika virus RNA extraction and detection via qRT-PCR

Samples were thawed on ice and 900 μL of BA-1 media was added to each tube before they were triturated using a Tissuelyser at 20,000 Hz (Qiagen, Hilden, Germany) for 2 min [28]. RNA was extracted from samples using a Thermo Scientific KingFisher Flex (Thermo Fisher Scientific, Waltham, MA) and the 5X MagMAX^™^—96 Viral RNA Isolation Kit (Applied Biosystems, Foster City, CA) [28]. Zika virus nucleic acid was detected with a LightCycler 96 (Roche Molecular Systems, Basel, Switzerland) using the SuperScript^™^ III Platinum^™^ One-Step qRT-PCR kit (Invitrogen, Carlsbad, CA) and a previously described primer-probe set targeting the Zika virus NS5 protein coding region [28, 29]. Bodies of all samples were analyzed first followed by the legs and wings and saliva of samples with bodies positive for Zika virus RNA.

To confirm that negative samples were truly negative and not simply below the level of detection by the qRT-PCR assay, negative samples were inoculated onto confluent Vero cells in 6-well tissue culture plates. Culture media was collected at days 1 and 5 post inoculation, extracted and again assayed by qRT-PCR to look for growth in titer indicative of replication (and thus infectiousness).

### DNA extraction and detection of *Wolbachia spp*. via PCR and gel-electrophoresis

As the endosymbiont *Wolbachia* is known to make *Aedes* mosquitoes refractory to Zika virus infection we tested for the presence of *Wolbachia* DNA in our samples [30]. Homogenate from ten mosquito bodies of each species were pooled into four groups of five. DNA was extracted using Qiagen’s DNeasy Blood and Tissue Kit according to the manufacturer’s instructions (Qiagen, Hilden, Germany). Extracted material was amplified by PCR using Qiagen’s HotStarTaq DNA Polymerase kit according to the manufacturer’s instructions using previously described primers (Qiagen, Hilden, Germany) [31]. PCR product was then run on a 2% agarose gel stained with SYBR safe in TAE at 120 mV for 45 min (Thermo Fisher Scientific, Waltham, MA). Gel was imaged using a Bio-Rad GelDoc (Bio-Rad, Hercules, CA).

## Results

Our data indicate that *Aedes aegypti* and *albopictus* mosquito populations in New Orleans, Louisiana are not competent vectors for Zika virus given low levels of viremia (Table 1). Only 1 *Ae*. *aegypti* was positive at 4 and 15 DPI while no *Ae*. *albopictus* were positive. None of the exposed mosquitoes had virus present in the saliva, indicating a low likelihood of successful establishment of Zika virus from low-level viremia and/or asymptomatic cases.

**Table 1:** Infection, dissemination and transmission results for New Orleans F3 *Ae*. *aegypti* (AAEG) and *Ae*. *albopictus* (ALBO) mosquitoes orally challenged with Zika-Panama virus.

Species	DPE	Total N	Bodies Positive	Legs/Heads Positive	Positive Saliva
AAEG	4	39	1	0	0
AAEG	10	29	0	0	0
AAEG	15	39	1	0	0
ALBO	4	22	0	0	0
ALBO	10	20	0	0	0
ALBO	15	22	0	0	0

Testing of the *in vitro* cell culture supernatants by qRT-PCR detected no Zika virus growth either by cytopathic effect or by qRT-PCR testing of the supernatant, even in the case of the two positive bodies. This indicates that the individuals originally categorized as positive via molecular testing were positive for viral RNA and not for infectious virus. PCR and gel-electrophoresis showed no evidence of *Wolbachia* infection in either pools of *Ae*. *aegypti* or *Ae*. *albopictus* tested.

## Discussion

Populations of *Ae*. *aegypti* and *Ae*. *albopictus* have been shown to be competent vectors of dengue virus and Zika virus both in the laboratory and field [32–36]. The competence of *Aedes* mosquitoes for transmitting Zika virus has been shown to be affected by such factors as species, mosquito and virus strain [37]. In addition to the infection and transmission rates, the extrinsic incubation period (EIP), or the time it takes for an individual mosquito to become capable of transmitting virus after taking an infectious blood meal, is known to be dependent on the multiplicity of infection, or the infectious dose of virus in a blood meal, and the number of subsequent blood meals taken.

The reported results of vector competence studies of *Ae*. *aegypti* and *Ae*. *albopictus* after oral challenge with a Zika virus-infectious blood meals have yielded mixed results regarding the magnitude of competency. Roundy *et al*. (2017) found that Cambodian and Mexican strains of Zika virus were less infectious in mosquitoes than a Senegalese strain [38]. They also found that blood-meals from live viremic donor mice were more infectious to mosquitoes than those from artificially prepared meals [38]. Furthermore, Tesla *et al*. (2018) found there is a significant correlation between viral titer of the blood-meal and temperature as well as EIP [39, 40]. Most recently, González *et al*. (2019) concluded there is limited risk of Zika virus transmission by Spanish populations of *Ae*. *albopictus* after they found no infectious virus in the saliva of orally challenged mosquitoes [41].

We hypothesized that New Orleans mosquitoes would be at best moderately competent for Zika virus at a dose representing low-viremia cases, given that no autochthonous transmission occurred in New Orleans at the height of the Zika virus epidemic in the Western Hemisphere. Our data indicates that these *Aedes* populations are not competent vectors for Zika virus, though there was some limited susceptibility to infection by a Panama strain of Zika virus. While there is limited chance for transmission given our data, there is the possibility that Zika virus would be detected in mosquito pools by surveillance efforts as a very small number of *Ae*. *aegypti* did become infected [28].

Our contemporaneous plaque assay controls demonstrated viable virus in each of the oral challenge blood meals, indicating that infectious virus was presented to the mosquitoes during challenge. One potential explanation for the low competence of New Orleans *Ae*. *albopictus* in particular, could have been the presence of the endosymbiotic bacterium *Wolbachia*, which is known to block the transmission of multiple mosquito-borne pathogens including Zika virus and occur in wild *Ae*. *albopictus* populations [30, 42]. We tested our mosquitoes for the presence of *Wolbachia* using previously published PCR primers and found no evidence that *Wolbachia* were present in the New Orleans populations [31]. Thus, we conclude that there are other mechanisms that underly the lack of susceptibility of New Orleans *Aedes* mosquitoes to this strain of Zika virus. Such mechanisms may include mosquito-virus kinetics, the level of viremia needed for this virus strain to establish an infection in *Aedes* mosquitoes, the ability of this Zika virus strain to establish and shed in the salivary glands of *Aedes* mosquitoes or the fact this Zika virus strain was isolated from a human sample and only ever passaged in Vero cells.

Further, mosquito populations can vary in vector competence across temporal scales [43], which could indicate that in the future, New Orleans mosquito populations may have different vector competence profiles. In addition, *Ae*. *aegypti* are often partial feeders which means they could feed more than once during a gonotrophic cycle. Thus, regardless of the low susceptibility in New Orleans mosquito populations, *Ae*. *aegypti* behavior may confer a slight advantage in relative competence as vectors compared to *Ae*. *albopictus*. In fact, *Ae*. *aegypti* and *Ae*. *albopictus* competence for Zika virus has been studied, and in general, *Ae*. *aegypti* is usually found to be more competent than *Ae*. *albopictus* [44].

## Conclusions

We observed remarkably low susceptibility of both *Ae*. *aegypti* and *Ae*. *albopictus* from New Orleans to a Zika virus strain from Panama after oral challenge with virus doses similar to that of asymptomatic or subclinical infections. These results suggest a limited risk of Zika virus transmission in the New Orleans area due to the silent infections of returning travelers. This likely played a major role in why there was not local transmission in New Orleans compared to Florida and Texas during the 2016 epidemic, despite multiple known travel associated introductions to New Orleans [15]. Despite these results, New Orleans *Aedes* spp. mosquito populations have been shown to be competent vectors for dengue, and thus control measures should not be relaxed.

## Supporting information

S1 Table Summary of oral challenge experiments. (DOCX)

S1 Data (XLSX)
