# Supplementary material for: Short Report: Asymptomatic Zika virus infections with low viral loads not likely to establish transmission in New Orleans Aedes populations
Source: PLoS One. 2020 May 29;15(5):e0233309. doi: 10.1371/journal.pone.0233309 (PMC7259492; doi:10.1371/journal.pone.0233309)
Supplement: S1 Table — (DOCX) [file pone.0233309.s001.docx]

**Table S1. Summary of oral challenge experiments.**

| **Meal PFU/mL** | ***Ae. aegypti*** **Engorged** | ***Ae. albopictus* Engorged** | **Cull Days** |
| --- | --- | --- | --- |
| 2.16 x 10^4^ | 124/300 (41.33%) | 68/300 (22.67%) | 4, 10, 15 |
